# Evaluation of Cannabis-Related Product Use Among Patients With Hidradenitis Suppurativa: A Narrative Review

**DOI:** 10.1177/12034754241266125

**Published:** 2024-07-31

**Authors:** Delaram Shojaei, Haleh Zabihi, Vincent Maida, Mark G. Kirchhof, Afsaneh Alavi

**Affiliations:** 1 Faculty of Medicine, University of British Columbia, Vancouver, BC, Canada; 2 Temerty Faculty of Medicine, University of Toronto, Toronto, ON, Canada; 3 Division of Palliative Care, University of Toronto, Toronto, ON, Canada; 4 Department of Dermatology, Faculty of Medicine, University of Ottawa and the Ottawa Hospital, Ottawa, ON, Canada; 5 Department of Dermatology, Mayo Clinic, Rochester, MN, USA

**Keywords:** medical dermatology, treatment response, substance use, clinical research, patient care

## Abstract

The use of cannabis and cannabis-related products among patients with hidradenitis suppurativa (HS) is increasing globally. Given the potential anti-inflammatory, therapeutic, and pain management benefits of cannabis-related products, we reviewed primary literature to evaluate the prevalence and possible purpose for cannabis use among patients with HS and to provide recommendations to patients and physicians. A narrative review of original studies was conducted using Embase and Ovid Medline databases. The search strategy was confirmed by a librarian and conducted on September 1, 2023, using a detailed list of subject headings and keywords tailored to cannabis, cannabis-related products, HS, and both adult and pediatric populations. Among 43 identified studies, 6 met the eligibility criteria and encompassed 34,435 patients. Patients were mostly female, and studies were conducted across the United States, Canada, and France. Findings show higher cannabis use among HS patients, demonstrating efficacy in pain management, sleep, anxiety relief, itch relief, and improved quality of life. Cannabis may play a role in analgesia, improved quality of life, pain, itch, and overall mental health in patients with HS and healthcare providers including dermatologists should increase their familiarity in appropriate use of cannabis-related products.

## Overview

Cannabis use among dermatology patients has garnered significant attention following its legalization in Canada and several states in the United States. Patients with hidradenitis suppurativa (HS) report a high prevalence of cannabis or cannabis-derived product consumption for both medical and recreational purposes. Recognizing these usage patterns and increasing clinician awareness is vital for holistic HS care; thus, this study aims to review the available literature on cannabis usage among HS patients and provide recommendations for patients and healthcare professionals in appropriate use of cannabis-related products.

## Characteristics of Cannabis-Related Products

Cannabis is a genus of flowering plants from the family Cannabaceae. *Cannabis sativa*, the most prevalent species, contains over 80 different types of cannabinoids, together with other important groups of chemicals such as terpenes and flavonoids.^1^ The most extensively studied cannabinoids in the cannabis plant are cannabidiol (CBD) and tetrahydrocannabinol (THC).^2^ Different products are available with varying concentrations of CBD and THC and formulations such as topical creams and oils, oral formulations such as food and snack items, and inhalation products such as cigarettes and vapes.^3^ Common side effects of CBD include fatigue, diarrhea, and changes in appetite, while THC may contribute to addiction, abnormal brain development, depression or anxiety, and lung disease.^4^ The Food and Drug Administration has approved several synthetic cannabinoids including Epidiolex (cannabidiol), Marinol and Syndros (dronabinol), and Cesamet (nabilone) which can be accessed with a medical prescription. Although these products have been approved for medical use, the negative side effects must also be considered and any potential harm must be outweighed by the benefits.

## Current Evidence on Cannabis-Related Product Use in Dermatology and HS

Studies indicate a variety of uses for cannabis in HS management, including analgesia, improved quality of life, pleasure, and benefits for pain, itch, and overall mental health through various formulations of cannabis-related products (Supplemental Tables S1 and S2).^5^ The observed anti-inflammatory properties of cannabis may contribute to its potential in expediting healing for HS patients.^6^ In addition, other reported purposes include itch relief and sleep benefits.^7^ The collective evidence from these studies underscores the potential role of cannabis as an adjunctive treatment in managing HS; however, caution is encouraged as the current literature on safety and benefits of cannabis-related products is limited and studies are heterogeneous.

### Therapeutic Management of HS

Cannabis use, particularly in topical formulations, has been positively correlated with the perceived improvement of several dermatological conditions, with demonstrated efficacy in the treatment of atopic dermatitis and acne.^8^ A survey by Mahurin and Maier reported that 10.6% of dermatology patients using cannabis or cannabinoid products used them to treat their condition, with HS ranking among the top 3 conditions for cannabis use.^9^

The diversity of formulations patients use in HS disease treatment is crucial. Topical formulations are relatively new and their long-term adverse effects are not yet known. Short-term use of inhalational cannabis products has been linked to psychomotor and cognitive impairment, diarrhea, vomiting, and somnolence,^10^ while chronic use increases the risk of oropharyngeal and lung cancers.^11,12^ Thus, when developing management plans for patients with HS, careful consideration of the benefits and potential harms of cannabis is essential, taking into account various concentrations and formulations.

### Pain Management

Chronic pain is a frequent and debilitating symptom of HS, and even the highest rated pain management modalities are only considered moderately effective by HS patients.^13^ Pain influences the psychosocial well-being and quality of life of patients and is commonly undertreated in HS, with 75.9% of patients reporting that they did not receive pain management recommendations from their healthcare provider.^13^ Only 12% of professionally managed HS patients are prescribed cannabinoids as a pharmacologic analgesic for chronic HS pain.^14^ Lesort et al found a statistical link between cannabis use and pain scores of HS patients during remission, suggesting that chronic pain associated with HS might play a role in cannabis addiction.^15^ Thus, self-treatment for pain using cannabis is a common practice in this patient population.^7^ Garg et al explored the dual role of cannabis in various forms in alleviating pain and enhancing the quality of life for HS patients with an existing substance use disorder (SUD).^16^ The study indicated that approximately 29.7% of HS patients with a SUD gravitated toward cannabis-related products, underscoring its potential as an adjunctive pain therapy.^16^

Among cannabis-related products as pharmacological analgesics prescribed by healthcare professionals, Fernandez et al predominantly described oral and inhaled products, with a consumption rate of 32.9% among HS patients.^13^ The preferred formulations included smoking (36.8%), edibles (11.1%), and a combination of both (52.1%). Effectiveness ratings for pain management were markedly higher for cannabis smoking and edibles compared to conventional analgesics like ibuprofen and acetaminophen (*P* < .0001). Although the efficacy of cannabis use did not significantly surpass that of opioids, the use of combination pain management options should be considered in an attempt to better control pain in HS patients.^13^

### Psychological Management

Patients with HS experience significant physical, emotional, and psychological impairment, which may explain the finding that this population group has a higher risk for depression, anxiety, suicidality, and a 50% greater odds of SUD.^16-18^ The prevalence of SUD was found to be higher among patients with HS (4%) compared to patients without HS (2%) and 29.7% of SUD diagnoses were attributed to cannabis.^16^ Given that cannabis-related products have been associated with reduced feelings of anxiety and depression, it is possible that patients with HS utilize cannabis as a psychological treatment and relief. Lesort et al also examined the motivations and demographics of cannabis product use among HS patients, and revealed a common purpose of seeking pleasure.^19^ Their findings indicate that HS patients exhibited a higher proclivity toward cannabis use compared to their counterparts with psoriasis, independent of Hurley and Dermatology Life Quality Index scores. These findings signify a broader cultural or psychological association with cannabis consumption, which must be taken into account when these products are considered in management of patients.

## Current Perspective and Attitude Toward Cannabis-Related Products

The widespread frustration of HS patients with conventional therapies propels patients toward exploring alternative therapies, including cannabis-based inhalation, oral, and topical options.^20^ Patients also feel that current management guidelines, particularly for HS pain management, are insufficient, and there is strong interest in understanding more about the potential for cannabis. Mahurin et al reported that 90% of patients expressed an interest to learn more about cannabis-related products, while 47.6% sought guidance from dermatologists regarding their application for skin conditions.^9^ Given that patients identified dermatologists as their preferred source of information, we highlight an excellent opportunity for patient education in clinical settings.^9^ According to Price et al, a substantial percentage of patients further find specific forms of cannabis-related products highly beneficial, with the preferences leaning toward smoking, oral CBD oil, and topical CBD oil.^20^ Efforts to reduce the stigma of utilizing cannabis-related products for medical purposes, education, and open discussion about the harms of illicit substances with patients are highly encouraged.

## Recommendations for Cannabis-Related Product Use for HS Symptom Management

**Figure 1.**
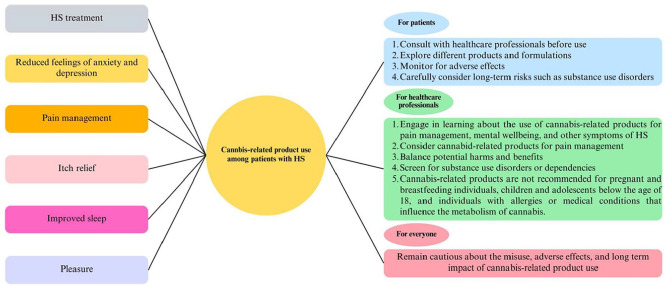
Visual diagram of evidence-based guidelines for utilizing cannabis-related products by patients and healthcare professionals.

Based on our findings, we have made recommendations for patients, healthcare professionals, and both (Table 1 and Figure 1).

**Table 1.** Evidence-Based Recommendations for the Use of Cannabis-Related Products Among Patients and Healthcare Professionals.

Healthcare stakeholders	Recommendations	Level of evidence
For patients	Consult with healthcare professionals when considering cannabinoid-related products as patients with HS may be at an increased risk for SUD and mental illness.^8,14,19^	4
	Explore different forms of cannabinoid products, including topical creams, oils, oral formulations, and inhalation products as there may be differences based on individual preferences, convenience, cost, and targeted area of treatment.	
	Regularly monitor for any adverse effects, including changes in skin condition, mental health, and overall well-being.^8,9,13^	
	Engage in careful consideration of the side effects and the long terms risks of cannabis-related products.^19^	
For healthcare professionals	Education of dermatologists about the prescription of cannabis-related products in adjunctive management of pain, mental well-being, and other symptoms in HS patients.^14,21^	
	Consider more effective pain medications using cannabis-related products in adjunct with other analgesics, regardless of the specific Hurley stage.^13,14^	
	Evaluate the potential harms and benefits of cannabinoid-related products in alleviating HS-related symptoms and improving quality of life in patients with HS.^14,16,19^	
	Younger, male patients with lower BMI may be more likely to utilize cannabis; Screen for development of SUDs or dependencies on cannabis related products.^16^	
	The use of cannabis-related products is not recommended for the following patient populations with HS: • Pregnant and breastfeeding individuals, due to the possible risks that may be imposed on a developing fetus or infant.^22^ • Individuals with sensitivity or allergies specific to cannabis-related products.^22^ • Individuals with specific medical conditions that may affect the metabolism of cannabinoids or increase risk of adverse effects (eg, individuals with SUD, psychosis, etc).^22,23^ • Children, due to the lack of well-established safety guidelines of cannabis related products for dermatological use in pediatric patients.^24^ • Adolescents <18 years old, due to the increased risk of adverse health outcomes if cannabis use initiates during adolescence.^22,23^	
For patients and healthcare professionals	Caution is encouraged when promoting the use of cannabis-related products as this review’s evidence of research is limited and the findings are heterogenous with respect to formulations, route of administration, dosage, and frequency of use.	N/A

Abbreviations: HS, hidradenitis suppurativa; SUD, substance use disorder.

## Conclusion

Cannabis and cannabis-related products come in different psychogenic forms with CBD and THC. CBD may cause fatigue and appetite changes, while THC may lead to addiction and long-term elevated risk of cancer. However, FDA-approved synthetic cannabinoids have risks and benefits. Chronic HS pain remains undertreated, which may contribute to an increased use of cannabis-related products over conventional analgesics for pain management among HS patients. These products may also be used for psychological relief of anxiety and depression, improve sleep, and provide itch relief. As a result, patients and healthcare professionals are encouraged to increase their awareness about the harms, benefits, and appropriate use of cannabis-related products to optimize health outcomes.

## Supplemental Material

sj-docx-1-cms-10.1177_12034754241266125 – Supplemental material for Evaluation of Cannabis-Related Product Use Among Patients With Hidradenitis Suppurativa: A Narrative Review Supplemental material, sj-docx-1-cms-10.1177_12034754241266125 for Evaluation of Cannabis-Related Product Use Among Patients With Hidradenitis Suppurativa: A Narrative Review by Delaram Shojaei, Haleh Zabihi, Vincent Maida, Mark G. Kirchhof and Afsaneh Alavi in Journal of Cutaneous Medicine and Surgery

sj-docx-2-cms-10.1177_12034754241266125 – Supplemental material for Evaluation of Cannabis-Related Product Use Among Patients With Hidradenitis Suppurativa: A Narrative Review Supplemental material, sj-docx-2-cms-10.1177_12034754241266125 for Evaluation of Cannabis-Related Product Use Among Patients With Hidradenitis Suppurativa: A Narrative Review by Delaram Shojaei, Haleh Zabihi, Vincent Maida, Mark G. Kirchhof and Afsaneh Alavi in Journal of Cutaneous Medicine and Surgery
