# Supplementary material for: Evaluation of Cannabis-Related Product Use Among Patients With Hidradenitis Suppurativa: A Narrative Review
Source: J Cutan Med Surg. 2024 Jul 31;28(5):481–5. doi: 10.1177/12034754241266125 (PMC11528859; doi:10.1177/12034754241266125)
Supplement: sj-docx-1-cms-10.1177_12034754241266125 – Supplemental material for Evaluation of Cannabis-Related Product Use Among Patients With Hidradenitis Suppurativa: A Narrative Review [file sj-docx-1-cms-10.1177_12034754241266125.docx]

| **Study authors (year)** | **Country** | **Number of patients** | **Age, mean (SD) or age group (%)** | **Sex** | **Study objective** |
| --- | --- | --- | --- | --- | --- |
| Fernandez et al (2022)^13^ | USA | 438 | 37.6 ± 10.7 | 93.8% women | Identify pain management therapies used in HS and assess patient perceived effectiveness |
| Garg et al (2018)^16^ | USA | 32,625 | 18-44: 61.1%  45-64: 38.9% | 76% women | Evaluate substance use disorder among patients with HS |
| Lesort et al (2019)^19^ | France | 641 | 32.9 ± 10.1 | 36% women | Determine the prevalence and reasons for cannabis use in patients with HS |
| Mahurin et al (2020)^9^ | USA | 210 | 18-29: 20.3%  30-44: 30.1%  45-60: 26.4%  > 60%: 21.7% | Not reported. | Prevalence and methods of cannabis/cannabinoid use among dermatology patients |
| Price et al. (2020)^20^ | USA, Canada | 303 | 38.0 ± 10.9 | 88.4% women | Explore patterns of conventional and CAM therapies used by patients with HS |

Table S1. Demographic and Clinical Characteristics of Hidradenitis Suppurativa Study Populations
