# Supplementary material for: Evaluation of Cannabis-Related Product Use Among Patients With Hidradenitis Suppurativa: A Narrative Review
Source: J Cutan Med Surg. 2024 Jul 31;28(5):481–5. doi: 10.1177/12034754241266125 (PMC11528859; doi:10.1177/12034754241266125)
Supplement: sj-docx-2-cms-10.1177_12034754241266125 – Supplemental material for Evaluation of Cannabis-Related Product Use Among Patients With Hidradenitis Suppurativa: A Narrative Review [file sj-docx-2-cms-10.1177_12034754241266125.docx]

Table S2. Usage and Effectiveness of Cannabis and Cannabinoids in Hidradenitis Suppurativa Studies

| **Study Authors (Year)** | **Primary purpose of usage (%)** | **Patient population** | **Prevalence of cannabis consumption (%)** | **Preferred formulation** | **Other reported outcomes** | **Level of evidence** | **Grade of Research** |
| --- | --- | --- | --- | --- | --- | --- | --- |
| Fernandez et al (2022)^13^ | Analgesic (100%) | Patients with an HS diagnosis | 32.9% | Smoking (36.8%)  Edibles (11.1)  Both (52.1%) | Cannabis usage increased with higher Hurley scores, but not statistically significant (Hurley stage I, 8.2%; II, 53.4%; and III, 38.4%)  24.1% reported receiving recommendations for HS pain management from a healthcare provider.  Effectiveness ratings for pain management significantly higher for cannabis smoking and edibles compared to ibuprofen and acetaminophen (p<0.0001)  Effectiveness ratings for pain management not significantly higher for cannabis use compared to opioids | IV | B |
| Garg et al (2018)^16^ | Analgesic | Patients with HS diagnosis and substance use disorder | 29.7% | Not reported. | Prevalence of substance use disorder among HS patients was 4% compared to 2% for patients without HS.  29.7% of substance use disorder among HS patients was in the form of cannabis related products. | IV | B |
| Lesort et al (2019)^19^ | Increased pleasure | Patients with an HS diagnosis  Patients with a psoriasis diagnosis | HS patients: 34%  Psoriasis patients: 11.6% | Not reported. | HS patients are at a higher risk for cannabis use than Psoriasis patients (P <0.001; odds ratio 285, 95% CI 151–564)  Cannabis use was statistically associated with VAS pain score during remission.  Cannabis use did not differ among varying Hurley scores or DLQI scores.  Cannabis users were more often men, younger, with a lower BMI (P < 0.01)  A vast majority of cannabis users in the HS group began cannabis use before disease onset (69.4%,)  Patients with a higher DLQI score were more likely to report pain (P < 0.001), stress (P = 0.010), and moral support (P < 0.001) as a motivation for cannabis use | IV | D |
| Mahurin et al.(2020)^9^ | Treatment (10.6%) | Patients with a dermatological condition including HS | 38.2% | Topical cream and oil | 90% of patients expressed interest in learning more about cannabis related products.  47.6% of patients expressed interest in seeing a dermatologist to obtain information about the use of cannabis-related products for their skin condition. | IV | Not applicable. |
| Price et al (2020)^20^ | Frustration with conventional treatment (63.9%)*  Desire to try a “new” (51.0%) or “more natural” treatment (44.3%)* | Patients with an HS diagnosis | 82.5% | Inhalation (33.3%)**  Oral CBD oil (26.4%)**  Topical CBD oil (23.1%)** | 57.3% of patients perceived marijuana, and 44.8% perceived topical cannabidiol oil as the most helpful. | IV | B |

* Most common reasons for patients to use any complementary and alternative treatment, including but not limited to cannabis and cannabis related products.

** Percentage is approximated as provided data is in qualitative format.
